# An educational intervention for medical students to improve self-efficacy in firearm injury prevention counseling

**DOI:** 10.1186/s40621-019-0201-3

**Published:** 2019-05-29

**Authors:** Jacky Z. Kwong, Jennifer M. Gray, Lisa Rein, Ying Liu, Marlene D. Melzer-Lange

**Affiliations:** 1 Department of Pediatrics – Section of Emergency Medicine, Medical College of Wisconsin, 8701 W Watertown Plank Rd, Milwaukee, WI 53226 USA; 2 Department of Biostatistics, Medical College of Wisconsin, 8701 W Watertown Plank Rd, Milwaukee, WI 53226 USA

**Keywords:** Firearm, Medical students, Intervention, Education

## Abstract

**Background:**

Most physicians support counseling patients about firearm injury prevention (FIP), but infrequently do so due to lack of training and low confidence. Interventions to increase counseling frequency should focus on improving physician self-efficacy. Firearm injuries affect many clinical specialties; therefore, trainees would benefit from early FIP education. This study aims to determine if a 20-min educational intervention improves self-efficacy in FIP counseling in third-year medical students. Knowledge and beliefs were also assessed as secondary indicators of self-efficacy.

**Methods:**

This was a prospective study performed at a medical school associated with a tertiary care children’s hospital during the 2016–17 academic year. Groups of 12–15 different third-year medical students were selected to receive either a 20-min intervention or control lecture during their monthly pediatric lectures. The intervention consisted of two clinical vignettes, a brief discussion about the importance of FIP, and suggestions for clinical integration. The control session was a case-based lecture about pediatric emergencies. Participants completed baseline electronic assessments. Intervention students also completed post-intervention assessments immediately following each session. All participants completed final assessments at 6 months. Data were analyzed using Wilcoxon signed-rank tests and Wilcoxon rank-sum.

**Results:**

We surveyed a total of 130 students. Sixty-five students completed the entire series of assessments – 22 from the control and 43 from the intervention group. There were no significant differences between the control and intervention groups at baseline. Immediately after, intervention, participants reported feeling more self-efficacious, had improved knowledge of FIP risk factors, and had beliefs more consistent with providing FIP anticipatory guidance (*p* <  0.001 for all three measures). After 6 months, participants sustained improvement in one of two self-efficacy questions (“I feel ready to counsel patients about firearm injury prevention”) and retained knowledge of risk factors (*p* <  0.05 for both). However, their beliefs did not significantly favor FIP counseling, and they were not more likely to engage in a conversation about firearm safety.

**Conclusions:**

A 20-min educational intervention acutely improved self-efficacy in FIP counseling in third-year medical students, but improvements weakened after six months. Without further training, the beneficial effects of a one-time intervention will likely wane with time.

## Background

In 2016, firearm injuries were the leading cause of death for 15- to 24-year-olds in the US (Web-based injury statistics query and reporting system 2018). Both fatal and non-fatal injuries impose a significant economic burden on society and devastate families and healthcare institutions (Lee et al. 2014; Fowler et al. 2015; Russo et al. 2016). Providing care to patients with firearm injuries is often multifaceted and involves multiple disciplines within healthcare (Avraham et al. 2018; Bayouth et al. 2018; Tasigiorgos et al. 2015; Greenspan and Kellermann 2002; DiScala and Sege 2004).

Most physicians agree on the need to address firearm injuries and believe that physicians should have an active role in prevention counseling (Betz et al. 2013; Grossman et al. 1995; Roszko et al. 2016; Solomon et al. 2002; Cassel et al. 1998). However, despite overwhelming support, physicians still do not regularly counsel their patients about firearm injury prevention (FIP) (Solomon et al. 2002; Cassel et al. 1998; Olson et al. 2007). In fact, most physicians reported low confidence in FIP counseling and inadequate or low rates of FIP training demonstrating an important deficiency in medical education (Butkus and Weissman 2014; Cheng et al. 1999; Finch et al. 2008; Price et al. 1997a; Price et al. 1997b; Price et al. 2010; Khubchandani et al. 2009).

Physician self-efficacy in counseling is related to the likelihood of counseling behaviors in practice and specifically appears to be a key determinant of FIP counseling (Solomon et al. 2002; Cheng et al. 1999; Finch et al. 2008). Physician beliefs and knowledge about FIP are also important components that influence and inform counseling behaviors, and have been studied as elements that directly contribute to self-efficacy (Abraham et al. 2001; Dingeldein et al. 2012).Therefore, educational efforts aimed to improve counseling behavior and practices should focus on improving self-efficacy in providing anticipatory guidance as well as supportive knowledge content.

Several studies have been published investigating FIP training and intervention outcomes (Abraham et al. 2001; Dingeldein et al. 2012; Barkin et al. 2008a; Hamilton et al. 2014). Though improvements in self-efficacy were reported, these studies were limited to pediatric residents. Given the broad scope of firearm injuries, including FIP training in undergraduate medical education may be ideal.

Our study aims to determine if a 20-min educational intervention improves FIP counseling in third-year medical students. In addition to reported counseling behavior, we assess student self-efficacy as our primary indicator of counseling behavior. We also evaluate student beliefs and knowledge as secondary indicators contributing to self-efficacy. Our intervention focuses on limiting children’s access to firearms by advocating for proper firearm storage as a means of reducing injuries (Grossman et al. 2005; Shenassa et al. 2004; Barkin et al. 2008b).

## Methods

**Fig. 1:**
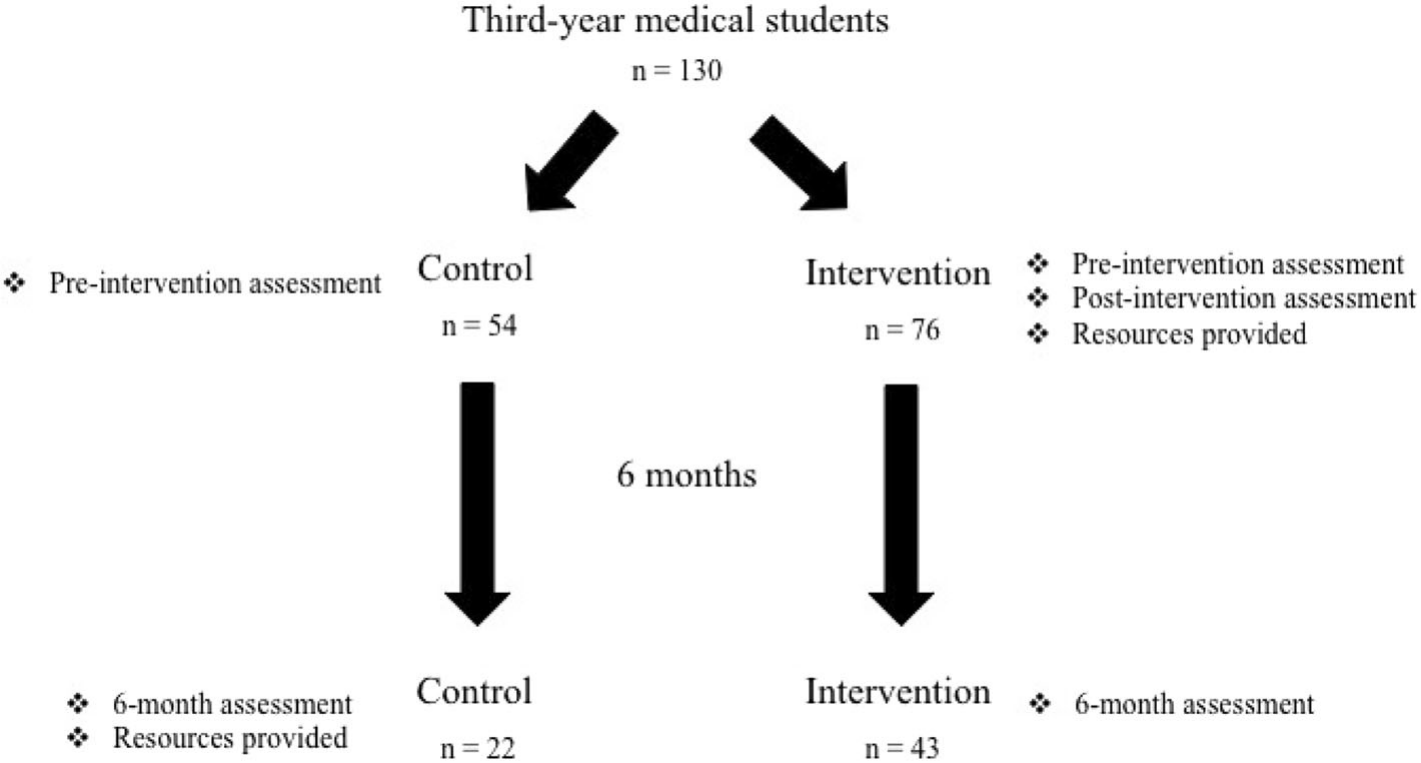
Study design and timing of assessments

This was a prospective, interventional study performed during the 2016–17 academic year at an academic medical school. Groups of 12–15 different third-year medical students attended a monthly lecture series during their pediatric clerkship rotation (Fig. 1). Each month, students received either a 20-min intervention or control lecture, depending on the scheduled speaker for that month. The intervention was developed and presented by medical students in conjunction with an attending pediatric emergency medicine physician. The intervention consisted of two clinical vignettes, a summary of FIP legislation and epidemiology, a brief discussion about the importance of FIP, and suggestions for clinical integration, including recommendations from the ASK campaign (www.askingsaveskids.org). Students who received the intervention were also given cable gun locks, provided by Master Lock™ (Model #107KADSPT, Milwaukee, WI) in partnership with the Emergency Nurses Association, at the end of the presentation. Locks were provided to students to encourage them to engage in a conversation about FIP with someone and give them the lock. Each lock came with a standard instruction handout. Participants in the intervention group were also provided with additional web-based resources immediately after the intervention. The control lecture was a standard case-based lecture about pediatric emergencies. Participants in the control group were also provided with the same web-based resources after completion of their 6-month assessments.

Study design and assessments were based on a previous study by Dingeldein et al. with alterations to coordinate with clerkship schedules and time limitations (Dingeldein et al. 2012). All study participants completed baseline electronic pre-intervention assessments. Participants in the intervention group also completed post-intervention assessments immediately following the session. All participants completed 6-month follow-up assessments to evaluate long-term effects of the intervention. Six-month follow-up assessments were sent to participants’ institutional emails six months after each corresponding lecture. A maximum of three emails were sent to participants to collect 6-month assessments. Participation in the intervention was voluntary, and all students were given the opportunity to opt out, but were encouraged to participate. Investigators were provided with the number of students scheduled to attend each lecture by the clerkship coordinator, but, all students who opted out or were absent could not be identified. All assessments were created and disseminated via SurveyMonkey™ (San Mateo, CA).

**Fig. 2:**
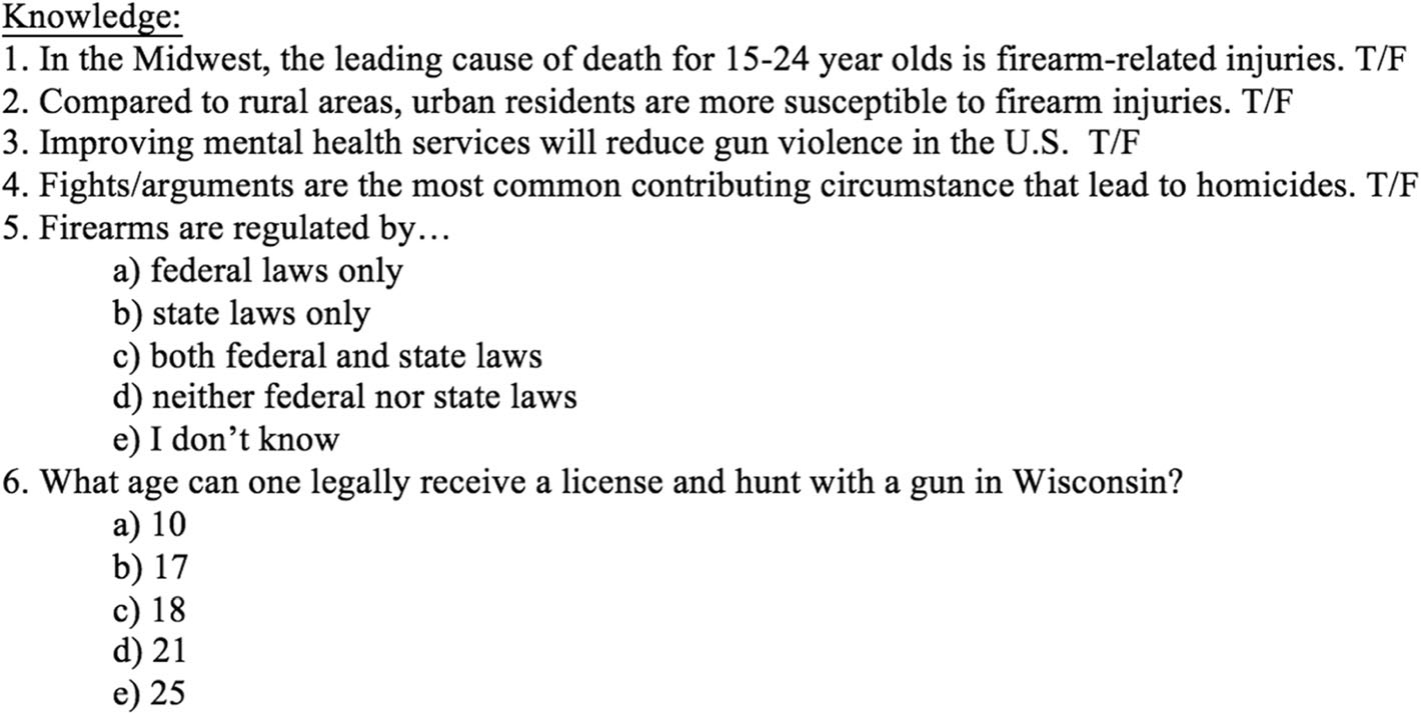
Knowledge-based questions included in pre-, post-, and 6-month assessments

All assessments evaluated participants’ perceived self-efficacy about FIP counseling, beliefs about FIP, and knowledge of risk factors as contributory indicators of FIP counseling behavior. Self-efficacy and belief questions used a 5-point Likert scale (1 = strongly disagree, 5 = strongly agree) while knowledge was evaluated by a 6-question survey (Fig. 2). Basic demographic information, previous experience with firearms, and participants’ institution emails were also collected. Institutional email addresses were collected in all assessments for tracking responses for statistical analysis and disseminating follow-up material (6-month assessments and web-based resources). Institutional email addresses often contained surnames of participants, but care was taken to minimize any other potentially identifying information as well as to maintain anonymity of their responses. Finally, 6-month follow-up assessments asked all students if they had a conversation about firearms with someone in the past 6 months. This study was approved by the Institutional Review Board at the Medical College of Wisconsin.

### Statistical analysis

Patterns of survey completion were reported. For all statistical tests, missing data were excluded via list-wise deletion, which assumes missing values are missing completely at random. Descriptive statistics were calculated for all demographic characteristics and assessment responses including mean, median, standard deviation, and range for continuous variables and frequency and percent for categorical variables. Baseline participant characteristics and assessment responses were compared between control and intervention groups using Wilcoxon rank-sum tests for continuous variables and Fisher’s exact tests for categorical variables. For the intervention group, pre- and post-intervention assessment responses and total quiz score were compared using Wilcoxon signed-rank tests. Pre- and 6-month assessment responses and total quiz scores were compared within control and intervention groups using Wilcoxon signed-rank tests. The change in scores (6-month – Pre) were compared between control and intervention groups using Wilcoxon rank-sum tests. All statistical analyses were performed using R version 3.3.1 (R Foundation for Statistical Computing, http://www.R-project.org). All p-values were 2-sided and p less than 0.05 was considered statistically significant.

## Results

We surveyed a total of 130 out of 139 students—54 from the control and 76 from the intervention—scheduled to attend the lecture series from the Class of 2018. Nine students either opted out or were absent on the day of the lecture and did not complete assessments. Of the 130, two from the control and one from the intervention were excluded immediately due to incomplete pre-session assessments. At baseline, there were no significant differences between the control and intervention group in age, gender, home state, or previous experience with firearms. Similarly, there were no significant differences in FIP self-efficacy, beliefs, or knowledge between the two groups (Table 1).

**Table 1:** Demographics of control and intervention groups prior to intervention

	All (*n* = 127)	Control (*n* = 52)	Intervention (*n* = 75)	p-value
Age				0.058
Mean (SD)	26.4 (2.5)	26.9 (3.1)	26.0 (1.9)	
Gender				0.370
M	72 (56.7%)	32 (61.5%)	40 (53.3%)	
F	55 (43.3%)	20 (38.5%)	35 (46.7%)	
Home State (collapsed)				0.858
WI	60 (47.2%)	24 (46.2%)	36 (48.0%)	
Other	67 (52.8%)	28 (53.8%)	39 (52.0%)	
Previous experience?				0.281
YES	71 (55.9%)	26 (50.0%)	45 (60.0%)	
NO	56 (44.1%)	26 (50.0%)	30 (40.0%)	

### Immediate effects of the intervention

Of the 75 students who completed the pre-intervention assessments, four students did not complete post-intervention assessments. The remaining 71 students from the intervention group were included in this analysis. Prior to the intervention, students did not feel ready to counsel patients or confident in providing advice and resources about firearm safety (mean = 2.9 and 3.0 respectively). (Table 2) Immediately after the intervention, students felt significantly more self-efficacious in both questions (*p* <  0.001 for both; mean difference = + 1.0 and + 1.1 respectively) (Table 3).

**Table 2:** Measures of self-efficacy, attitude/beliefs and knowledge of FIP in control and intervention groups prior to receiving educational intervention*

	All (*n* = 127)	Control (*n* = 52)	Intervention (*n* = 75)	p-value
1. I feel ready to counsel patients about firearm injury prevention				0.984
Mean (SD)	2.9 (1.1)	2.9 (1.1)	2.9 (1.1)	
2. I am confident I can provide appropriate advice and resources to patients about firearm safety				0.360
Mean (SD)	2.7 (0.9)	2.6 (0.8)	2.7 (0.9)	
3. There is not enough time in a doctor visit to talk about injury risk and prevention with a patient				0.885
Mean (SD)	3.1 (1.1)	3.0 (1.1)	3.1 (1.0)	
4. Asking patients about firearms is a violation of privacy and can damage the patient-doctor relationship				0.474
Mean (SD)	1.8 (0.7)	1.8 (0.8)	1.8 (0.7)	
5. Physicians should be trained to provide firearm safety counseling				0.778
Mean (SD)	3.8 (0.8)	3.8 (0.9)	3.9 (0.7)	
6. Gun violence should be considered a public health issue				0.235
Mean (SD)	4.4 (0.8)	4.5 (0.8)	4.4 (0.7)	
7. It is the physician’s role to counsel and advise about firearm safety and prevention				0.277
Mean (SD)	3.6 (0.9)	3.5 (0.8)	3.7 (0.9)	
8. Number of correct quiz responses				0.842
Mean (SD)	3.0 (1.2)	2.9 (1.2)	3.0 (1.2)	

*Questions 1–2 = self-efficacy, 3–7 = attitudes/beliefs, 8 = knowledge

**All responses were scored using a 5-point Likert scale (1 = strongly disagree, 5 = strongly agree)

**Table 3:** Measures of self-efficacy, attitude/beliefs and knowledge of FIP in intervention groups before and immediately after receiving educational intervention*

*n* = 71	Pre	Post	Diff	*p*-value
1. I feel ready to counsel patients about firearm injury prevention				< 0.001
Mean (SD)	2.9 (1.1)	3.9 (0.7)	1.0 (1.0)	
2. I am confident I can provide appropriate advice and resources to patients about firearm safety				< 0.001
Mean (SD)	2.8 (1.0)	3.9 (0.6)	1.1 (0.9)	
3. There is not enough time in a doctor visit to talk about injury risk and prevention with a patient				< 0.001
Mean (SD)	3.1 (1.0)	2.7 (1.0)	−0.4 (0.8)	
4. Asking patients about firearms is a violation of privacy and can damage the patient-doctor relationship				0.849
Mean (SD)	1.8 (0.6)	1.8 (0.8)	0.0 (0.6)	
5. Physicians should be trained to provide firearm safety counseling				< 0.001
Mean (SD)	3.9 (0.7)	4.2 (0.7)	0.3 (0.6)	
6. Gun violence should be considered a public health issue				0.458
Mean (SD)	4.4 (0.7)	4.5 (0.6)	0.0 (0.5)	
7. It is the physician’s role to counsel and advise about firearm safety and prevention				< 0.001
Mean (SD)	3.7 (0.9)	4.0 (0.8)	0.3 (0.7)	
8. Number of correct quiz responses				< 0.001
Mean (SD)	3.0 (1.1)	5.4 (1.0)	2.4 (1.3)	

*Questions 1–2 = self-efficacy, 3–7 = attitudes/beliefs, 8 = knowledge

** *All responses were scored using a 5-point Likert scale (1 = strongly disagree, 5 = strongly agree)*

There were five questions assessing beliefs about FIP in the context of medical practice. Pre-assessment results demonstrated that student beliefs favored FIP counseling and education in all five questions at baseline. Immediately after the intervention, responses to three of the five questions shifted even more towards favoring FIP (*p* <  0.001 for all three questions). The remaining two questions, evaluating beliefs about violation of patient privacy and gun violence as a public health issue, remained unchanged. Students still disagreed asking patients about firearms is a violation of patient privacy (mean = 1.8) and still agreed gun violence should be considered a public health issue (mean = 4.5) after the intervention (Table 3).

There were six questions assessing knowledge of FIP risk factors. Prior to the intervention, students averaged 3.0 correct out of 6.0. Immediately after the intervention, scores increased significantly to a mean of 5.4 out of 6.0 (*p* <  0.001) (Table 3).

### 6-month effects of the intervention

Sixty-five students were included in this analysis – 22 from the control and 43 from the intervention. Sixty-five students were excluded due to failure to complete any of the three assessments. After 6 months, students who received the intervention expressed increased readiness to counsel patients about FIP compared to students who received the standard lecture (*p* <  0.05). Additionally, students who received the intervention had improved scores of FIP risk factors (*p* <  0.05) (Table 4).

**Table 4:** Measures of self-efficacy, attitude/beliefs and knowledge of FIP in intervention groups before and 6 months after receiving educational intervention*

	Control (*N* = 22)				Intervention (*N* = 43)				Comparison of diff’s
	Pre	6mo	Diff	Paired *p*-value	Pre	6mo	Diff	Paired *p*-value	*p*-value
1.I feel ready to counsel patients about firearm injury prevention				0.745				< 0.001	0.022
Mean (SD)	3.0 (1.1)	3.1 (1.1)	0.1 (1.1)		3.0 (1.1)	3.9 (0.7)	0.9 (1.3)		
2. I am confident I can provide appropriate advice and resources to patients about firearm safety				0.115				< 0.001	0.105
Mean (SD)	2.6 (1.0)	2.9 (1.1)	0.3 (0.9)		2.9 (1.0)	3.6 (0.7)	0.7 (0.9)		
3. There is not enough time in a doctor visit to talk about injury risk and prevention with a patient				0.182				0.260	0.415
Mean (SD)	2.8 (1.2)	3.3 (1.1)	0.5 (1.8)		2.9 (0.9)	3.1 (0.9)	0.2 (1.1)		
4. Asking patients about firearms is a violation of privacy and can damage the patient-doctor relationship				0.212				0.851	0.216
Mean (SD)	1.7 (0.8)	1.9 (0.8)	0.2 (0.8)		1.8 (0.6)	1.7 (0.6)	−0.0 (0.7)		
5. Physicians should be trained to provide firearm safety counseling				0.107				0.004	0.913
Mean (SD)	3.8 (0.9)	4.2 (0.8)	0.3 (1.1)		3.8 (0.7)	4.1 (0.6)	0.4 (0.8)		
6. Gun violence should be considered a public health issue				0.020				0.660	0.052
Mean (SD)	4.7 (0.5)	4.5 (0.8)	−0.3 (0.5)		4.4 (0.6)	4.5 (0.7)	0.0 (0.7)		
7. It is the physician’s role to counsel and advise about firearm safety and prevention				0.212				0.196	0.857
Mean (SD)	3.7 (0.8)	4.0 (0.6)	0.3 (0.9)		3.7 (0.9)	3.8 (0.8)	0.2 (0.8)		
8. Number of correct quiz responses				0.128				< 0.001	0.018
Mean (SD)	2.9 (1.4)	3.2 (1.2)	0.4 (1.0)		2.9 (1.1)	4.1 (1.1)	1.3 (1.5)		

* *Questions 1–2 = self-efficacy, 3–7 = attitudes/beliefs, 8 = knowledge*

**All responses were scored using a 5-point Likert scale (1 = strongly disagree, 5 = strongly agree)

However, students no longer felt as confident in providing advice and resources about firearm safety. Furthermore, though their beliefs still favored FIP counseling and education in medical practice, there were no significant differences in any of the five belief-type questions when compared to students in the control group after 6 months. Of note, students’ beliefs about the statements “Asking patients about firearms is a violation of privacy and can damage the patient-doctor relationship” and “Gun violence should be considered a public health issue” were already one-sided at baseline. Students who received the intervention were also not more likely to engage in a conversation about firearm safety or storage (Table 5). In fact, the majority of students, who received a gun lock as part of the intervention, did not give the gun lock to anyone (78.6%).

**Table 5:** Measure of FIP centered conversations had by control and interventional groups at 6 months

	Control (*n* = 22)	Intervention (*n* = 43)	p-value
In the past 6 months, have you had a conversation about firearm safety or storage with someone?			0.268
NO	17 (77.3%)	26 (60.5%)	
YES	5 (22.7%)	17 (39.5%)	

## Discussion

According to the social learning theory (previously social cognitive theory), one’s self-efficacy, knowledge, and beliefs are important determinants of one’s behavior, particularly in the context of health-related behaviors (Bandura 2004). This theory has been studied to target educational interventions for both patients and physicians (Mohebi et al. 2013; Ha et al. 2018; Ozer et al. 2004; Maiuro et al. 2000). Of note, it has specifically shown improvements in FIP counseling (Solomon et al. 2002; Finch et al. 2008; Abraham et al. 2001; Dingeldein et al. 2012). However, it is also important to note the intrinsic limitations of the theory and recognize the complexity of the determinants of counseling.

Our intervention was successful in maintaining improvements in the self-efficacy question about feeling ready to conduct FIP counseling after six months. We postulate that the regression observed in the second question (“I am confident I can provide appropriate advice and resources to patients about firearm safety”) is most likely due to the lack of repetition and subsequent degredation in behavior practice and self-efficacy. In a review by Bailey et al. summarizing neurobiological findings, long-term potentiation, defined as repeated stimulation and conditioning of a certain behavior, was pivotal in the formation of long-term memory from short-term memory (Bailey et al. 2015). Educational data further support the benefits of practice and repetition in memory retention, particularly spaced repetition (Kang 2016). Alternatively, without repeated stimulation, hippocampal pruning of short-term memory will puruse resulting in the loss of behavioral pattern (Bailey et al. 2015). In summary, the old adage – “use it or lose it” – likely explains the loss of participants’ self-efficacy.

The intervention showed categorical retention of knowledge of FIP at 6 months. This retention of knowledge is encouraging and may have directly contributed to improvement of participants’ self-efficacy. However, participants’ beliefs about FIP did not persist after 6 months. Immediately after the intervention, we observed significant changes in three of the five belief questions. These changes were no longer significant during follow-up. The question about gun violence being a public health issue likely saw no difference because the baseline response was high agreement at 4.4/5. However, they were still consistent with other studies examining physician opinions about FIP and favor physician involvement (Betz et al. 2013; Grossman et al. 1995; Roszko et al. 2016; Solomon et al. 2002; Cassel et al. 1998).

Gun locks were provided to students in the intervention group in response to Barkin et al.’s observation that FIP counseling behavior may have decreased due to the diminishing availability of locks for patients (Barkin et al. 2008a). Since gun locks were not provided to the control group, no conclusion can be drawn about whether the participants given gun locks were more likely to engage in FIP counseling than those who did not.

As previously mentioned, the broad scope of firearm injuries may lend itself to ideal placement in undergraduate medical education (UME). However, we would advocate for including FIP training at multiple levels at difference time points of medical education from UME to attending level continued medical education (CME) courses. Providing anticipatory guidance for firearm injuries is supported by multiple professional medical organizations including the American Academy of Pediatrics and the American College of Surgeons (AMA Recommends New, Common-sense Policies to Prevent Gun Violence 2018; Dowd and Sege 2012; Statement on Firearm Injuries 2013; Butkus et al. 2014). The benefits of spaced repetition has also been demonstrated by several studies showing increased likelihood of physician FIP counseling if provided continued training (Price et al. 2007; Kaplan et al. 1998). Without periodic reinforcement, physician and physician trainee self-efficacy in providing FIP counseling will likely diminish with time, as seen in our study.

This study has several important limitations. First and most notably, our study lost a significant number of students to six-month follow-up. Of the original 130 students, only 65 students (50%) followed through and completed the full series of surveys. Attendance for the didactic was required by the clerkship, but participation in our study was voluntary. Six-month assessments were also distributed without regard for holiday breaks and exams. Additionally, students participated in these sessions throughout the duration of their third year. It is likely that clinical experience of each student at the time of intervention could not be controlled for; and therefore, students may have felt more or less comfortable having discussions with families as the academic year progressed. Third, endpoint data were collected at 6 months and our results were not validated. As mentioned before, further studies should investigate more distant time points and validate their results by objectively measuring student behavior. Finally, our second self-efficacy question (“I am confident I can provide advice and resources…”) was a double-barrelled statement combining two elements (advice and resources) into a single question, which may have influenced the non-improvement seen in our results. It is also important to note that this educational intervention was performed in Milwaukee, where exposure to firearm violence is prevalent, and may influence student beliefs and open-mindedness to our intervention.

## Conclusions

A 20-min educational intervention acutely improved self-efficacy in FIP counseling in third-year medical students, but improvements weakened after six months. Students retained knowledge of FIP risk factors after six months and had beliefs consistent with previously reported clinician attitudes about FIP. But without further training, the beneficial effects of a one-time intervention will likely wane with time. Including FIP training into medical school curricula may encourage development of more competent and effective physicians.

## Abbreviations

CME: Continued medical education
FIP: Firearm injury prevention
UME: Undergraduate medical education
